# The Influence of Different Smoking Procedures on the Content of 16 PAHs in Traditional Dry Cured Smoked Meat “Hercegovačka Pečenica”

**DOI:** 10.3390/foods8120690

**Published:** 2019-12-17

**Authors:** Leona Puljić, Krešimir Mastanjević, Brankica Kartalović, Dragan Kovačević, Jelena Vranešević, Kristina Mastanjević

**Affiliations:** 1 The Faculty of Agriculture and Food Technology (APTF) of the University of Mostar, Biskupa Čule bb, 88000 Mostar, Bosnia and Herzegovina; leonapuljic224@gmail.com; 2 Faculty of Food Technology Osijek, Josip Juraj Strossmayer University of Osijek, F. Kuhača 20, 31000 Osijek, Croatia; dragan.kovacevic@ptfos.hr (D.K.);; 3 Scientific Veterinary Institute Novi Sad, Rumenački put 20, 21000 Novi Sad, Serbia; brankica@niv.ns.ac.rs (B.K.); vet.bmv@hotmail.com (J.V.)

**Keywords:** PAH content, Hercegovačka pečenica, traditional smoking, industrial smoking

## Abstract

During smoking, meat products may get contaminated by polycyclic aromatic hydrocarbons (PAH), especially the ones that are smoked in traditional (uncontrolled) conditions. This study aims to evaluate the difference in PAH content in samples of traditional dry cured pork meat products, “Hercegovačka pečenica”, produced in (1) a traditional smokehouse and (2) in industrial chambers. The study revealed that the content of the four priority PAHs (PAH4) in samples produced in a traditional smoking manner highly exceeded (up to 10 times) the maximal limits set for PAHs (12 µg/kg). PAH4 in all samples subjected to industrial smoking procedures was below the limit of quantification. All samples had below-the-limit-of-quantification values for Benzo[a]pyrene. The surface layer of the samples produced in traditional conditions had the highest total content of PAH16. The inner parts of all samples, whether traditional or industrial, had significantly lower PAH16 concentration than the surface layer.

## 1. Introduction

Hercegovačka pečenica is a traditional dry cured pork meat product produced in Herzegovina (a southern region of Bosnia and Herzegovina) in a specific way. Smoked pork loin is a cured meat product obtained from a long back muscle lat, musculus longissimus dorsi, without bones and skin, using a salting or brining process. Other spices or herbs may be added optionally. The smoking, drying, and ripening of the product takes from one to three months. Specific geographical conditions of Herzegovina (the influence of the sub-Mediterranean climate in particular) with a significant influence of the Mediterranean and in some parts of the continental climate, distinguishes Hercegovačka pečenica from similar products originating from other areas. The characteristic of this microclimate is bora and sirocco winds, which meet and collide only in this area. The peculiarity of this product is the smoke of hornbeam and beech trees from the high altitudes of the nearby mountains. Meat smoking is widely used in the production of traditional dry cured meat products from Herzegovina. One of these is the traditional Hercegovačka pečenica. A typical diet in Herzegovina involves different kinds of smoked meat products. The direct smoking during the drying process is still the most commonly used technique with traditional producers.

The production of Hercegovačka pečenica mainly takes place in smokehouses on family farms. Traditional smoking involves treating of pre-salted pork loins with wood smoke. During smoking, particular sensorial features in terms of taste, color, and aroma are formed (phenol derivatives, carbonyls, organic acids, among others) [1,2,3]. In addition, smoking improves conservation due to its dehydrating, bactericidal, and antioxidant properties [2]. Incomplete wood combustion during the process of smoking is responsible for the production of significant amounts of polycyclic aromatic hydrocarbons (PAHs), a potential health hazard [4,5]. They have two or more fused aromatic rings and are known as cancer-causing agents [6]. PAHs differ in their carcinogenicity. Even though some of them are regarded as non-carcinogenic, they may increase the carcinogenicity of other PAHs [7]. PAHs are highly lipophilic, and thus can be found in meat products subjected to smoking [8,9,10]. The factors that limit the occurrence of PAHs during the smoking process are various. The most important ones are the following: the technique of smoking, choice of wood, the duration of smoke exposure, and the type of food itself [11,12,13]. Codex alimentarius [14] guidelines for optimal smoking should be applied to reduce the PAH concentrations in processed foods.

The European food safety authority (EFSA) decided that the concentrations of benzo[a]pyrene (BaP) and the sum of the concentrations of four PAHs: benzo[a]pyrene (BaP), benz[a]anthracene (BaA), benzo[b]fluoranthene (BbF), and chrysene (Chry) (PAH4) [15], will be considered a reference for determination of PAHs in food. According to Bosnian and Herzegovinian regulations on maximum levels for certain contaminants in food [16], complied with the European commission (EU) regulation no. 835/2011 [17], the maximum permissible concentration of BaP in meat products is set at 2 μg/kg and the sum of PAH4 concentrations should not exceed 12 μg/kg. Although there are studies across Europe and some developing countries concerning the carcinogenic potential and occurrence of PAHs in food products [1,5,10,18,19,20,21,22], there is a lack of information about PAH occurrence in meat products and smoked meat products, in particular in Bosnia and Herzegovina. Thus, the aim of this study was to investigate and compare the differences in PAH content in the samples of “Hercegovačka pečenica” subjected to traditional and industrial smoking processes.

## 2. Materials and Methods

### 2.1. Preparation of Smoked Meat Samples

Raw material processing is performed using traditional technology. The samples were made from pork long back muscle lat, musculus longissimus dorsi. Immediately before salting, the weight of each individual raw loin was determined by weighing (approximately 3 kg), followed by salting with a 50:50 mixture of rock and nitrite salt. The meat was salted once manually by adding an indefinite amount of salt over it. The meat was then left in the salt for seven days in a cooling chamber at a temperature of 4 °C. After the salting processes were completed, the loins were washed in water and transferred to a drying and smoking room where they were drained and tempered for the next 12 to 20 h. The description of smoking conditions is shown in Table 1.

When the traditional smoking method was applied, raw and pre-salted pork loins (three replicates) were put at three meters distance from open fire. The smoking was carried out by combustion of dry hard wood (beech) every day for the first six days (for 6–8 h), and every two or three days (for 2–3 h) for the next fourteen days. It lasted for 20 days in ambient conditions. The temperature ranged from 3.5 to 11.2 °C (average 6.9 °C) and relative humidity from 61.3 to 90.5% (average 74.2%).

In industrial production, pre-salted pork loins (three replicates), were kept in a ripozo chamber for 13 days at the average temperature of 1 °C and relative humidity of 60% until they lost about 15% of the initial weight. A smoking chamber (Mauting, Czech Republic) was used for smoking in industrial production. The chamber was equipped with a heating plate smoke generator where beech sawdust was placed. During industrial smoking, the average temperature was 19.0 °C and average relative humidity was 74.4%. The pork loins were smoked for 4 h a day (8 × 30 min) for three days.

After the smoking, pork loins were placed in a ripening chamber (Mauting, Valtice, Czech Republic). Drying and ripening was set at the average temperature of 15.0 °C and relative humidity was 74.2%. The sampling was carried out at the end of the smoking process (on the 20th day for when traditional procedure was used in and on the 3rd day in the case of the industrial). The total production time for both production procedures (industrial and traditional) was 45 days. All sampling was performed in triplicate. Before the PAH determination, the samples were packed in glass jars and stored in the dark at −30 °C until the analysis was performed, approximately 1 week after the sampling. All the analyses were done in triplicate.

### 2.2. GC-MS Analysis

Sample preparation for GC_MS analysis and chromatographic separation of 16 PAH (Nap—naphthalene; Anl—acenaphthylene; Ane—acenaphtene; Flu—fluorene; Ant—anthracene; Phen—phenanthrene; Flt—fluoranthene; BaA—benzo[a]anthracene; Pyr—pyrene; Chry—chrysene; BbF—benzo[b]fluoranthene; BkF—benzo[k]fluoranthene; BaP—benzo[a]pyrene; DahA—dibenzo[a;h]anthracene; BghiP—benzo[g;h;i]-perylene; InP—indeno[1;2;3-cd]pyrene) were conducted according to Mastanjević et al. [10]. The average values for precision, reproducibility, accuracy, linearity, LOQ, and LOD for PAH method validation can be found in Supplementary Table S1.

### 2.3. Statistical Analysis

Analysis of variance (ANOVA) and Fisher’s least significant difference (LSD), with significance defined at *p* < 0.05, were performed for all measured data. Statistica 12.7 (StatSoft Inc. Tulsa, OK, USA, 2015) was used for statistical analysis.

## 3. Results and Discussion

The raw material (pork loins) used for production of Hercegovačka pečenica contained only light PAHs (Nap, Anl, Ane, Flu, and Ant). The other eleven PAHs were below the limit of quantification. Ant was the PAH with the highest concentration of 21.6 µg/kg in raw meat. Other PAHs determined in raw pork loin samples ranged as follows: Nap 10.87 µg/kg, Anl 5.88 µg/kg, Ane 20.0 µg/kg, and Flu 5.46 µg/kg. In Slavonska kobasica, Mastanjević et al. [10] reported similar values for PAHs in raw materials. On the other hand, Djinovic et al. [23] reported lower values of PAHs in raw meat used for the production of different types of smoked meat products from Serbia. According to Ciganek and Neca (2006) [24], the PAHs found in animal tissues are a result of environmental contamination, thus the PAHs in raw pork meat in this research can be attributed to the contamination of feed used in pig breeding.

Mean values of PAHs determined in dry cured loins at the end of the smoking period (the 3rd and 20th day of production) are shown in Table 2. For all batches, PAHs contamination levels are presented for external/surface and inner parts.

PAH content determined in loins (external and inner parts) at the end of the smoking period in the samples subjected to traditional production (20 days of production, open fire) involved Nap, Anl, Flu, Ant, Phen, Flt, BaA, Pyr, BbF, BkF, and BghiP. On the other hand, PAHs determination in loins at the end of the smoking period in the samples subjected to industrial production (3 day production) resulted in NaP and Anl. The other analyzed PAHs were below the limit of quantification for all the sample groups. These results are in accordance with the previous research reports regarding smoked meat products [23,25], and different smoked sausages [2,10,26,27,28,29,30]. The most abundant light PAH in all samples appeared to be Phen, which ranged from 1029 µg/kg for batch TS-SP to <LOQ for batch IS-SP, showing a difference with statistical significance (*p* < 0.05) between batch TS-SP and all other sample groups. Groups TS-MP, IS-MP, and IS-SP did not show a difference with statistical significance for Phen content. Nap concentrations were between 26.7 µg/kg (TS-MP) and 35.5 µg/kg (IS-SP) and no difference with statistical significance was found between groups.

The content of Anl was between 21.2 µg/kg (TS-MP) and 590 µg/kg (TS-SP), showing a difference with statistical significance (*p* < 0.05) between TS-SP and all other sample groups. Flu concentrations ranged from below LOQ (IS-SP) to 406 µg/kg, (TS-SP), showing a difference with statistical significance (*p* < 0.05) between TS-SP and all other sample groups. Ant content ranged from below LOQ (IS-SP) to 242 µg/kg (TS-SP), showing a difference with statistical significance (*p* < 0.05) between TS-SP and all other sample groups. Flt concentrations ranged from below LOQ (IS-SP) to 82.7 (TS-SP) µg/kg with a difference with statistical significance (*p* < 0.05) detected between TS-SP and all other sample groups.

The results for BaA were between below LOQ (IS-SP) and 30.9 µg/kg (TS-SP) with a difference of statistical significance (*p* < 0.05) between all groups except between IS-MP and IS-SP. The content of Pyr was quantified below LOQ (IS-SP) to the maximum value of 54.5 µg/kg in sample TS-MP, with a significant difference (*p* < 0.05) between TS-SP and all other sample groups. BbF concentrations were in the range from below LOQ (IS-SP) to 1.54 µg/kg, (TS-SP) with the difference with statistical significance (*p* < 0.05) between traditional (TS-SP, TS-MP) and industrial (IS-SP, IS-MP) meat products.

The content of BkF was between below LOQ for IS-SP and 4.89 µg/kg in TS-MP. A significant statistical difference (*p* < 0.05) was noted between all groups except between IS-MP and IS-SP. BghiP concentrations were in the range from below LOQ (IS-SP) to 3.00 µg/kg, (TS-MP) and a significant statistical difference (*p* < 0.05) was detected between all groups except between IS-MP and IS-SP.

The sum of PAH16 ranged from 57.9 µg/kg for samples smoked in industrial conditions to 2474 µg/kg for samples smoked in traditional conditions. Mastanjević et al. [10] reported lower summation values of PAH16 for the samples at the end of the smoking phase, smoked in traditional conditions (509 μg/kg), but a higher sum of PAH16 (114 μg/kg) for samples subjected to industrial conditions. On the other hand, Djinovic et al. [23], reported lower PAH16 concentration in different smoked meat products from Serbia, measured at the end of the smoking process. PAH4 content ranged as follows: BaA < LOQ–30.9 μg/kg, Chry < LOQ, BbF < LOQ–1.54 μg/kg, BaP < LOQ, PAH4 < LOQ–32.5 μg/kg. In all sample groups, BaP content was lower than 2 μg/kg. The TS-MP and TS-SP samples had higher concentrations than the prescribed PAH4 content (12.7 μg/kg and 32.5 μg/kg).

The content of the four priority PAHs in all samples produced using traditional smoking techniques exceeded maximal limits set by Bosnian and Herzegovinian Regulation on maximum levels for certain contaminants in food [16], complied with the EC Regulation No. 835/2011 [17]. Such high amounts of PAH have rarely been reported before. However, it corresponds to the values reported for smoking under uncontrolled technological conditions, typical for households and developing countries [6,30]. On the other hand, the amount of the four priority PAHs in all the samples produced using industrial smoking procedures were < LOQ.

Sixteen PAHs in finished dry cured loins are presented in Table 3. The same PAH content (Nap, Anl, Flu, Ant, Phen, Flt, BaA, Pyr, BbF, BkF, and Bghip) was determined in traditional smoked “Hercegovačka pečenica” at the end of the production process. Contrary to this, in industrial smoked samples, only Nap and Anl were detected. The most abundant PAH in traditional smoked samples was Ant, where the content ranged from 2925 µg/kg in the surface of the product to 60.3 µg/kg in the inner part with difference with statistical significance of (*p* < 0.05) between groups. In industrially smoked samples, the most abundant PAH was Anl and it ranged from 3.80 µg/kg in the inner part of the product to 19.3 µg/kg on the surface and no difference with statistical significance (*p* > 0.05) between groups was detected. Nap concentrations were between 3.77 µg/kg (IS-MP) and 718 µg/kg (TS-SP), showing a difference with statistical significance (*p* < 0.05) between TS-SP and all other sample groups. The content of Anl was between 3.80 µg/kg (IS-MP) and 2515 µg/kg (TS-SP). The difference with statistical significance (*p* < 0.05) was detected between TS-SP and all other sample groups. Flu concentrations were in the range from <LOQ (IS-SP) to 701 µg/kg, (TS-SP) with the difference of statistical significance (*p* < 0.05) between TS-SP and all other sample groups. Phen content ranged from <LOQ (IS-SP) to 807 (TS-SP) µg/kg with the difference with statistical significance (*p* < 0.05) between TS-SP and all other sample groups. Flt concentrations ranged from <LOQ (IS-SP) to 237 µg/kg (TS-SP) with the difference with statistical significance (*p* < 0.05) between TS-SP and all other sample groups. BaA concentrations were between below LOQ (IS-SP) and 123 µg/kg (TS-SP) with difference with statistical significance (*p* < 0.05) between TS-SP and all other sample groups. The content of Pyr was between <LOQ (IS-SP) and 187 µg/kg (TS-SP) with difference with statistical significance (*p* < 0.05) between TS-SP and all other sample groups. BbF concentrations were in the range from <LOQ (IS-SP) to 2.36 µg/kg (TS-MP) showing a difference with statistical significance between traditional (TS-SP, TS-MP) and industrial (IS-SP, IS-MP) meat groups. The content of BkF was between <LOQ (IS-SP) and 6.43 µg/kg (TS-MP) with difference with statistical significance (*p* < 0.05) between all groups except between groups IS-MP and IS-SP. BghiP concentrations were in the range from <LOQ (IS-SP) to 2.79 µg/kg, (TS-MP) with difference with statistical significance (*p* < 0.05) between all groups except between the groups IS-MP and IS-SP.

External/surface parts of Hercegovačka pečenica smoked industrially and traditionally showed Σ 16 PAHs 25.6 µg/kg and 8225 µg/kg. The inner parts of all samples (both industrial and traditional) had a significantly lower total PAHs contamination levels (197 µg/kg for traditionally smoked samples and 7.57 µg/kg for industrial method of smoking). Ciecierska et al. (2007) [25] reported much lower concentrations of PAH 15 in both traditionally and industrially smoked raw cured loins (external part 10.7 µg/kg and inner part 1.52 µg/kg for traditional production and 9.42 µg/kg and 2.59 µg/kg for industrial production). Higher values for PAH content in Hercegovačka pečenica are probably a result of intensely extended smoking.

In general, the concentrations (in both external and inner parts) of all determined light PAHs at the end of the production process of Hercegovačka pečenica were higher than concentrations determined at the end of smoking. Mastanjević et al. [10] reported similar results for Slavonska kobasica. They presumed that this is due to the dehydration. At the end of the industrial production, the concentrations of all determined PAHs were lower than concentrations determined at the end of smoking.

The amount of the four priority PAHs in the samples produced by traditional smoking highly exceeded maximal limits set by Regulation (EU) No. 835/2011 (12 µg/kg) up to 10 times. In all samples produced by industrial smoking, the PAH4 content was below the limit of quantification. BaP concentration in investigated samples was below the limit of quantification. The highest total content of PAH16 (8225 µg/kg) was determined on the surface samples produced in traditional smokehouses at the end of the production. According to the results, one of the main factors which contributed to high levels of PAH in Hercegovačka pečenica is the smoking technique. Another important factor is smoking duration. The longer the samples are smoked, the higher concentration of the PAH can be expected [31,32]. In this research, the samples smoked in a traditional manner (open fire, 20 days of smoking) had significantly higher levels of 16 PAH content than industrially smoked samples. Also, the wood type used for smoking can significantly affect the PAH content in smoked meat products [2].

## 4. Conclusions

The use of traditional smoking method resulted in higher PAH contamination than the industrial. At the end of production, the inner parts of all smoked samples produced using both methods retained significantly lower total PAHs concentration, as well as less individual PAHs than the surface layer. The amount of the four priority PAHs in samples subjected to traditional smoking highly exceeded maximum limits set by the Regulation (EU) No 835/2011 (12 µg/kg) by up to 10 times. The consumption of this kind of products can be potentially harmful to human health and that is the reason why the ALARA (as low as reasonably achievable) principle is in force in the EU [33]. On the other hand, the amounts of the four priority PAHs in all samples subjected to industrial smoking processes were below the limit of quantification. The result of this study indicated that, in order to decrease the level of PAHs and reduce the risk of PAHs occurrence in smoked meat products, local producers should learn how to use the improved/novel smoking techniques and adjust the smoking parameters. This should result in safer smoked meat products.

## Figures and Tables

**Table 1:** Production conditions and variables of Hercegovačka pečenica samples.

Abbreviation	Batch	Smoking Time (days)	Sampling Position
TS-SP	Traditional smoking at 3 m distance from fire	20	surface
TS-MP	Traditional smoking at 3 m distance from fire	20	inner
IS-SP	Industrial smoking	3	surface
IS-MP	Industrial smoking	3	inner

**Table 2:** 16 PAHs (µg/kg) in Hercegovačka pečenica at the end of smoking process.

PAH	TS-SP	TS-MP	IS-SP	IS-MP
Nap	28.1 ^a^	26.7 ^a^	35.5 ^a^	30.9 ^a^
Anl	590 ^a^	21.2 ^b^	31.5 ^b^	26.9 ^b^
Ane	-*	-*	-*	-*
Flu	406 ^a^	16.0 ^b^	-*	-*
Ant	242 ^a^	8.71 ^b^	-*	-*
Phen	1029 ^a^	44.8 ^b^	-*	-*
Flt	82.7 ^a^	5.44 ^b^	-*	-*
BaA	30.9 ^a^	11.4 ^b^	-*	-*
Pyr	54.5 ^a^	2.48 ^b^	-*	-*
Chry	-*	-*	-*	-*
BbF	1.5 ^a^	1.29 ^a^	-*	-*
BkF	4.09 ^b^	4.89 ^a^	-*	-*
BaP	-*	-*	-*	-*
DahA	-*	-*	-*	-*
BghiP	2.77 ^b^	3.00 ^a^	-*	-*
Inp	-*	-*	-*	-*
∑PAH4	32.5 ^a^	12.7 ^b^	-*	-*
∑PAH16	2474 ^a^	145 ^b^	67.0 ^c^	57.9 ^d^

PAHs: polycyclic aromatic hydrocarbons; TS-SP: Traditional smoking at 3 m distance from fire (surface); TS-MP: Traditional smoking at 3 m distance from fire (inner); IS-SP: Industrial smoking (surface); IS-MP: Industrial smoking (inner). Means within rows with different superscripts are significantly different (*p* < 0.05); -*: < LOQ (limit of quantification); ∑PAH4: BaA (benzo[a]anthracene); Chry (chrysene); BbF (benzo[b]fluoranthene), and BaP (benzo[a]pyrene) ∑PAH16: Nap (naphthalene), Anl (acenaphthylene), Ane (acenaphtene), Flu (fluorene), Ant (anthracene), Phen (phenanthrene), Flt (fluoranthene), BaA, Pyr (pyrene), Chry, BbF, BkF (benzo[k]fluoranthene), BaP, DahA (dibenzo[a;h]anthracene), BghiP (benzo[g;h;i]-perylene), and InP (indeno[1;2;3-cd]pyrene).

**Table 3:** 16 PAHs (µg/kg) in the finished product Hercegovačka pečenica.

PAH	TS-SP	TS-MP	IS-SP	IS-MP
Nap	718 ^a^	54.8 ^b^	6.33 ^b^	3.77 ^b^
Anl	2515 ^a^	30.2 ^b^	19.3 ^b^	3.80 ^b^
Ane	-*	-*	-*	-*
Flu	701 ^a^	13.8 ^b^	-*	-*
Ant	2925 ^a^	60.3 ^b^	-*	-*
Phen	807 ^a^	11.6 ^b^	-*	-*
Flt	237 ^a^	5.79 ^b^	-*	-*
BaA	123 ^a^	5.41 ^b^	-*	-*
Pyr	187 ^a^	4.10 ^b^	-*	-*
Chry	-*	-*	-*	-*
BbF	2.25 ^a^	2.36 ^a^	-*	-*
BkF	4.38 ^b^	6.43 ^a^	-*	-*
BaP	-*	-*	-*	-*
DahA	-*	-*	-*	-*
BghiP	2.60 ^b^	2.79 ^a^	-*	-*
Inp	-*	-*	-*	-*
∑PAH4	125a	7.77b	-*	-*
∑PAH16	8225a	197b	25.6c	7.6d

TS-SP—Traditional smoking at 3 m distance from fire (surface); TS- MP—Traditional smoking at 3 m distance from fire (inner); IS-SP—Industrial smoking (surface); IS-MP—Industrial smoking (inner). Means within rows with different superscripts are significantly different (*p* < 0.05); -*: < LOQ (limit of quantification); ∑PAH4: BaA; Chry; BbF, and BaP ∑PAH16: Nap, Anl, Ane, Flu, Ant, Phen, Flt, BaA, Pyr, Chry, BbF, BkF, BaP, DahA, BghiP, and InP.

## Supplementary Materials

The following are available online at https://www.mdpi.com/2304-8158/8/12/690/s1, Table S1: The average values for precision, reproducibility, accuracy, linearity, LOQ and LOD for PAH method validation.
