# Supplementary material for: The Influence of Different Smoking Procedures on the Content of 16 PAHs in Traditional Dry Cured Smoked Meat “Hercegovačka Pečenica”
Source: Foods. 2019 Dec 17;8(12):690. doi: 10.3390/foods8120690 (PMC6963550; doi:10.3390/foods8120690)
Supplement: Supplementary file 1 [file foods-08-00690-s001.pdf]

foods

Table S1. The average values for precision, reproducibility, accuracy, linearity, LOQ and LOD for PAH method validation.

PAHs	Precision (%)	Reproducibility (%)	Accuracy (%)	Linearity (r^2)[a]	LOQ (µg/kg)	LOD (µg /kg)
Nap	11.3	6.33	95.0	0.99853	1.20	0.30
Anl	7.91	7.82	99.0	0.99672	1.30	0.29
Ane	8.52	8.32	99.3	0.99768	1.05	0.32
Flu	2.82	10.2	100	0.99792	1.11	0.30
Ant	3.53	3.73	98.7	0.99603	1.10	0.30
Phen	4.31	11.4	85.9	0.99847	1.18	0.35
Flt	3.61	3.72	95.3	0.99787	1.15	0.30
BaA	9.44	8.6	89.7	0.99825	1.30	0.37
Pyr	4.74	6.91	91.1	0.99792	1.21	0.32
Chry	5.33	8.20	92.5	0.99810	1.13	0.34
BbF	8.52	14.3	86.4	0.99408	1.30	0.36
BkF	3.51	3.32	94.3	0.99796	1.21	0.32
BaP	3.23	3.81	96.8	0.99871	2.00	0..53
DahA	8.72	11.3	91.2	0.99534	1.99	0.51
BghiP	9.71	11.3	81.5	0.99781	1.90	0.45
InP	9.51	10.3	85.3	0.99524	1.91	0.53
min	2.82	3.32	81.5	0.99853	1.05	0.30
max	11.3	14.3	100	0.99672	1.81	0.50

(r^2)[A]—Coefficient of determination; Nap—naphthalene; Anl—acenaphthylene; Ane—acenaphtene; Flu — fluorene; Ant — anthracene Phen — phenanthrene; ; Flt — fluoranthene; BaA — benzo[a]anthracene; Pyr — pyrene; Chry — chrysene; BbF — benzo[b]fluoranthene; BkF — benzo[k]fluoranthene; BaP—benzo[a]pyrene; DahA—dibenzo[a;h]anthracene; BghiP—benzo[g;h;i]-perylene; InP—indeno[1;2;3-cd]pyrene; LOD—limit of detection; LOQ—limit of quantification.
